# Inappropriate use of ritodrine hydrochloride for threatened preterm birth in Japan: a retrospective cohort study using a national inpatient database

**DOI:** 10.1186/s12884-019-2352-1

**Published:** 2019-06-18

**Authors:** Daisuke Shigemi, Shotaro Aso, Hideo Yasunaga

**Affiliations:** Department of Clinical Epidemiology and Health Economics, School of Public Health, The University of Tokyo, Tokyo, Japan

**Keywords:** Evidence, Practice gap, Threatened preterm birth, Tocolysis, Ritodrine hydrochloride

## Abstract

**Background:**

No study has revealed the effectiveness of long-term tocolysis for patients diagnosed with threatened preterm birth, and the use of betamimetics in these patients has not been recommended in the United States or Europe because of the potential for severe maternal adverse effects. However, long-term tocolysis with intravenous infusion of ritodrine hydrochloride, a betamimetic, can be selected as the first-line tocolytic treatment in Japan. This study was performed to (i) examine the current status of long-term tocolytic treatment, particularly with intravenous infusion of betamimetics, for threatened preterm birth in Japan and (ii) clarify the association between long-term tocolytic treatment and maternal adverse effects.

**Methods:**

This retrospective cohort study was conducted using a national inpatient database for acute-care inpatients in Japan. Among all pregnant women who were diagnosed with threatened preterm birth and admitted to the hospital from July 2010 to March 2016, we identified 134,959 eligible patients. The primary outcome was maternal serious adverse effects during hospitalization. A multivariable logistic regression analysis was performed to evaluate factors associated with maternal adverse effects.

**Results:**

Among all patients, 17.2% received intravenous infusion of ritodrine hydrochloride for ≤48 h and 28.7% received this treatment for ≥28 days. The proportion of maternal adverse effects was significantly higher among patients treated for ≥28 days than ≤48 h. A longer duration of tocolysis was significantly associated with increased maternal adverse effects.

**Conclusions:**

Long-term tocolysis was associated with an increased incidence of maternal adverse effects in the current study using real-world data. Japanese clinicians should adjust their tocolytic treatment practices in accordance with the latest scientific evidence or make efforts to verify the effectiveness and safety of long-term tocolysis.

## Background

An evidence–practice gap is defined as failure of physicians to adopt evidence-based best practices and may lead to poor outcomes for patients. Disparities between usual professional practice and evidenced-based guidelines have been seen in various clinical settings [1–3].

In the obstetrics field, preterm birth remains a major cause of perinatal morbidity and mortality and longitudinal disability [4]. When used for < 48 h, tocolytic agents can effectively provide sufficient time for antenatal glucocorticoid administration to delay delivery in the preterm birth period and improve neonatal outcomes [5]. However, studies have failed to show the effectiveness of long-term tocolysis [5, 6], and the United States Food and Drug Administration does not recommend long-term use of tocolytic agents. Use of betamimetics has not been recommended in the United States or Europe because of the potential for severe maternal adverse effects such as lung edema, granulocytopenia, and rhabdomyolysis [7–10].

However, a gap may exist between the available evidence and actual clinical practice for preterm birth in Japan. Japanese obstetricians may be likely to select long-term tocolysis with intravenous low-dose (50–200 μg/min) infusion of ritodrine hydrochloride, a betamimetic. The Japanese guideline does not exclude this type of practice, based on a previous limited study that insisted on the effectiveness of long-term (> 48 h) tocolysis for treating threatened premature birth [6]. However, the study had strong biases, and the current Japanese guidelines, published in 2017, cite no articles supporting the effectiveness of long-term tocolysis. The largest concern regarding such an unscientific practice is the possible increase in adverse outcomes for women, including venous thromboembolism resulting from a prolonged duration of bed rest and intravenous infusion. No changes in the Japanese perinatal guidelines have been made to reduce long-term tocolysis in the past 7 years.

Using a national inpatient database in Japan, the present study was performed to (i) examine the current status of long-term tocolytic treatment for threatened preterm birth among hospitalized women in Japan and (ii) clarify the association between long-term tocolytic treatment and adverse effects (lung edema, granulocytopenia, rhabdomyolysis, thromboembolism, and gestational diabetes mellitus). Furthermore, we discuss the possible reasons for this evidence–practice gap, including systems-, provider-, and patient-level barriers that are not amenable to adopting evidence.

## Methods

For this retrospective cohort study, we used the Diagnosis Procedure Combination database, a national inpatient database for acute-care inpatients in Japan. The details of this database are described elsewhere [11]. Briefly, about 1000 hospitals participate in the database and provide data for approximately 8 million inpatient admissions annually, representing about 50% of all acute-care inpatients in Japan. The attending physicians are required to accurately record the disease diagnoses because the diagnostic records are linked to a payment system that is based on medical insurance. The database includes the following data: dates of admission and discharge, patient age and sex, body weight and height, primary and secondary diagnoses, pre-existing comorbidities at admission and complications after admission, procedures performed, medications and devices used, in-hospital mortality, pregnancy status (pregnant or not), gestational age at admission, and delivery during hospitalization. Diagnoses, comorbidities, and complications are recorded using the International Classification of Diseases Tenth Revision (ICD-10) codes and text data in Japanese. The database contains no laboratory data or obstetric examinations (including the Bishop score, uterine cervical length, and vaginal bacteriological culture results).

We identified pregnant women aged 13 to 50 years who were diagnosed with threatened preterm birth from 22 to 36 weeks of gestation (ICD-10 code, O470) with complete data and admitted to the hospital from July 2010 to March 2016. Patients given intravenous magnesium sulfate as the initial tocolysis agent or oral nifedipine (calcium antagonist) for maintenance tocolysis were excluded.

Age was categorized into ≤19, 20–24, 25–29, 30–34, 35–39, and ≥ 40 years. The duration of intravenous administration of ritodrine hydrochloride was examined and categorized into ≤48 h, 3–6 days, 7–13 days, 14–27 days, and ≥ 28 days.

The primary outcome was maternal adverse effects (lung edema, granulocytopenia, rhabdomyolysis, thromboembolism, and gestational diabetes mellitus) during hospitalization.

Categorical variables (smoking, multiple births, premature rupture of membranes at admission, and placenta previa) were compared by the chi-square test or Fisher’s exact test. Continuous variables (age, body mass index, and gestational age at admission) were compared by a *t*-test or the Mann–Whitney U-test, as appropriate. A multivariable logistic regression analysis was performed to evaluate factors associated with maternal adverse effects. All statistical analyses were performed using IBM SPSS software version 23 (IBM Corp., Armonk, NY). All tests were two-tailed, and the threshold for significance was *P* < 0.05.

Written informed consent was not required because of the anonymous nature of the data. The need for written consent was formally waived by the ethics committee. The Institutional Review Board at The University of Tokyo approved the study. All authors obtained permission to use the national inpatient database.

## Results

During the study period, we identified 373,858 patients with a diagnosis of threatened preterm birth. Of these, we selected 134,959 eligible patients. The mean (standard deviation [SD]) age was 31.3 (5.4) years, and the mean (SD) gestational age was 30 (3.7) weeks.

The patient characteristics according to the duration of ritodrine hydrochloride infusion are shown in Table 1. The proportion of patients who underwent tocolytic treatment for ≤48 h was 17.2%, while the proportions who underwent tocolytic treatment for 3–6 days, 7–13 days, 14–27 days, and ≥ 28 days were 16.0, 16.2, 21.9, and 28.7%, respectively. Older women were more likely to receive tocolytic treatment for ≥28 days. Women with multiple births or placenta previa were likely to receive a longer duration of tocolytic treatment, while those with premature rupture of membranes at admission had a shorter duration of tocolytic treatment.

**Table 1:** Patient characteristics according to duration of ritodrine hydrochloride infusion

		Total	Duration of ritodrine hydrochloride infusion										*P* value
			≤48 h		3–6 days		7–13 days		14–27 days		≥28 days		
Number of patients		134,959	23,178	(17.2)	21,550	(16.0)	21,911	(16.2)	29,556	(21.9)	38,764	(28.7)	
Age in years, mean (SD)	< 20	2833	545	(19.2)	521	(18.4)	521	(18.4)	576	(20.3)	670	(23.6)	< 0.001
	20–24	12,399	2309	(18.6)	2213	(17.8)	2106	(17.0)	2755	(22.2)	3016	(24.3)	
	25–29	33,390	5657	(16.9)	5268	(15.8)	5451	(16.3)	7399	(22.2)	9615	(28.8)	
	30–34	46,216	7717	(16.7)	7209	(15.6)	7395	(16.0)	10,227	(22.1)	13,668	(29.6)	
	35–39	32,418	5522	(17.0)	5103	(15.7)	5223	(16.1)	6976	(21.5)	9594	(29.6)	
	≥40	7703	1428	(18.5)	1236	(16.0)	1215	(15.8)	1623	(21.1)	2201	(28.6)	
BMI, kg/m^2^	< 18.5	94,611	15,022	(15.9)	14,588	(15.4)	15,256	(16.1)	21,124	(22.3)	28,621	(30.3)	< 0.001
	18.5–24.9	6856	1182	(17.2)	983	(14.3)	1016	(14.8)	1366	(19.9)	2309	(33.7)	
	25.0–29.9	24,981	4938	(19.8)	4480	(17.9)	4230	(16.9)	5453	(21.8)	5880	(23.5)	
	≥30.0	5399	1306	(24.2)	1010	(18.7)	924	(17.1)	998	(18.5)	1161	(21.5)	
Smoking	No	109,253	18,309	(16.8)	17,172	(15.7)	17,815	(16.3)	24,195	(22.1)	31,762	(29.1)	< 0.001
	Yes	12,636	2305	(18.2)	2258	(17.9)	2073	(16.4)	2641	(20.9)	3359	(26.6)	
Multiple births	No	120,943	21,562	(17.8)	20,230	(16.7)	20,137	(16.6)	25,989	(21.5)	33,025	(27.3)	< 0.001
	Yes	14,016	1616	(11.5)	1320	(9.4)	1774	(12.7)	3567	(25.4)	5739	(40.9)	
PROM	No	130,527	21,338	(16.3)	20,527	(15.7)	21,351	(16.4)	29,054	(22.3)	38,257	(29.3)	< 0.001
	Yes	4432	1840	(41.5)	1023	(23.1)	560	(12.6)	502	(11.3)	507	(11.4)	
Placenta previa	No	126,431	22,187	(17.5)	20,451	(16.2)	20,545	(16.2)	27,235	(21.5)	36,013	(28.5)	< 0.001
	Yes	8528	991	(11.6)	1099	(12.9)	1366	(16.0)	2321	(27.2)	2751	(32.3)	
Gestational age at admission in weeks, mean (SD)		30 (3.7)	31.1	(3.9)	30.7	(4.0)	31.1	(3.7)	30.7	(3.1)	27.9	(2.9)	< 0.001

*BMI* body mass index, *PROM* premature rupture of membranes at admission, *SD* standard deviation

Table 2 shows the proportions of maternal adverse effects divided by the duration of ritodrine hydrochloride infusion. The proportion of maternal adverse effects was significantly higher for women who underwent tocolytic treatment for ≥28 days than ≤48 h (2.7% vs. 0.5%, respectively; *P* < 0.001). Maternal adverse effects among patients who underwent tocolytic treatment for ≤48 h included lung edema (*n* = 27, 23.4%), granulocytopenia (*n* = 3, 2.6%), rhabdomyolysis (*n* = 10, 8.7%), thromboembolism (*n* = 15, 13.0%), and gestational diabetes mellitus (*n* = 60, 52.2%). Among the women who underwent tocolytic treatment for ≥28 days, maternal adverse effects included lung edema (*n* = 25, 2.4%), granulocytopenia (*n* = 25, 2.4%), rhabdomyolysis (*n* = 39, 3.8%), thromboembolism (*n* = 275, 26.5%), and gestational diabetes mellitus (*n* = 673, 64.9%).

**Table 2:** Crude outcome: prevalence of maternal adverse effects and duration of ritodrine hydrochloride infusion

	Total	Duration of ritodrine hydrochloride infusion				
		≤48 h	3–6 days	7–13 days	14–27 days	≥28 days
Prevalence of maternal adverse effects^a^	1.4% (1927/134959)	0.5% (115/23178)	0.8% (164/21550)	1.0% (210/21911)	1.4% (401/29556)	2.7% (1037/38764)

^a^Composite outcome including any of the following: lung edema, granulocytopenia, rhabdomyolysis, thromboembolism, and gestational diabetes mellitus

The results of the multivariable logistic regression analysis are presented in Table 3. The occurrence of maternal adverse effects was significantly associated with older age, higher body mass index, placenta previa, and lower gestational age at admission. After adjustment for these variables, maternal adverse effects were significantly associated with a longer duration of ritodrine hydrochloride infusion.

**Table 3:** Multivariable logistic regression results for prevalence of maternal adverse effects^a^

	Odds ratio	95% CI			*P* value
Age (years)					
≥ 40	2.07	1.60	to	2.67	< 0.001
35–39	1.66	1.34	to	2.07	< 0.001
30–34	1.45	1.17	to	1.79	< 0.001
25–29	1.20	0.96	to	1.50	0.12
< 20	0.76	0.45	to	1.27	0.29
20–24 (reference)	1.00		to		
BMI (kg/m^2^)			to		
≥ 30.00	2.29	1.89	to	2.76	< 0.001
25.0–29.9	1.43	1.27	to	1.61	< 0.001
< 18.5	0.85	0.67	to	1.08	0.18
18.5–24.9 (reference)	1.00				
Smoking					
Yes	1.10	0.94	to	1.28	0.23
No	1.00				
Multiple births					
Yes	1.08	0.94	to	1.25	0.28
No	1.00				
PROM					
Yes	0.74	0.51	to	1.06	0.10
No	1.00				
Placenta praevia					
Yes	1.23	1.03	to	1.46	0.02
No	1.00				
Gestational age at admission	0.91	0.90	to	0.92	< 0.001
Duration of ritodrine hydrochloride					
≤ 48 h (reference)	1.00				
3–6 days	1.45	1.13	to	1.87	< 0.001
7–13 days	1.90	1.49	to	2.42	< 0.001
14–27 days	2.56	2.05	to	3.19	< 0.001
≥ 28 days	4.17	3.39	to	5.14	< 0.001

*BMI* body mass index, *PROM* premature rupture of membranes at admission, *CI* confidence interval

^a^Composite outcome including any of the following: lung edema, granulocytopenia, rhabdomyolysis, thromboembolism, and gestational diabetes mellitus

## Discussion

Among 134,959 women with threatened preterm birth treated with ritodrine hydrochloride, only 17.2% received treatment for ≤48 h. Long-term tocolytic treatment for ≥28 days accounted for 28.7% of patients. There was a significant association between a longer duration of tocolysis and maternal adverse effects. Advanced maternal age and obesity were also significantly associated with maternal adverse effects. Additionally, previous studies have indicated that advanced maternal age and obesity are related to preterm birth [12–14].

Betamimetics have not been recommended in the United States or Europe because of their risk of maternal adverse effects [7–10]. Furthermore, there is no strict evidence that long-term tocolysis has beneficial effects on reductions in neonatal morbidity and mortality [5]. One Japanese study showed no significant association between long-term tocolysis and improved outcomes (frequency of preterm delivery and neonatal outcomes) for threatened preterm birth [15]. Nevertheless, the present study revealed that long-term ritodrine hydrochloride infusion is commonly performed in Japan. The medications commonly administered globally for threatened preterm birth (including nifedipine, atosiban, and indomethacin) cannot be used for standard therapy in Japan because nifedipine is not included in the universal healthcare coverage for threatened preterm birth and atosiban is an unapproved drug in Japan. Additionally, indomethacin is contraindicated for pregnant women according to the package insert or prescribing information of indomethacin in Japan.

The present study suggests that a gap exists between the knowledge of what does and does not work based on the best available evidence and the clinical practice of treating threatened preterm birth in Japan. Although how this evidence–practice gap has eventuated remains unclear, we speculate on several possible reasons as described below.

One reason may be failure to overcome inertia. The latest version of the Japanese Perinatal Guideline 2017 states the following: “Long-term tocolysis above 48 hours is widely performed in Japan, and there are few negative articles on the therapeutic effect of the treatment. Therefore, long-term tocolysis under strict observation of adverse effects is one of the available options” [16]. Physicians always feel a need to do something for their patients, and few people ask questions of physicians. This situation may have resulted in physicians’ failure to overcome inertia [17].

Another reason may be related to patients and their family. Long-term tocolysis for preterm birth has gained popularity and become the de facto standard. This situation can affect patients’ expectation to receive the treatment because they know that the treatment is popular.

Data on the adverse effects of long-term tocolysis are lacking. Our results showed a cumulative increase in maternal serious adverse effects with a longer duration of tocolysis by low-dose ritodrine hydrochloride infusion. Long-term tocolysis may have led to prolonged catheter use and long bed rest with progression of gestational age, possibly resulting in a higher occurrence of adverse events. Thus, long-term tocolysis with intravenous ritodrine hydrochloride cannot be justified because of its uncertain effectiveness and the increased incidence of adverse effects.

Several limitations of this study should be acknowledged. This was a retrospective observational study based on an administrative database, and it lacked some clinical information such as parity, uterine cervical length as determined by ultrasound, testing of microorganisms in the vagina, and laboratory blood tests. The proportion of maternal adverse effects in this study was relatively low compared with that in previous studies [18–21]. This may be because of the limited components of the outcomes for maternal adverse effects and the low dosage of ritodrine hydrochloride. Additionally, we were unable to confirm the methods of diagnosis on threatened preterm birth in the current database. In Japan, criteria of diagnosis on threatened preterm birth are various among each hospital. Generally, short cervical length, frequent uterine contraction, uterine contraction with lower abdominal pain, or genital bleeding are typical reason for diagnosis of threatened preterm birth.

## Conclusions

In conclusion, the present study demonstrated that long-term tocolysis with ritodrine hydrochloride has been widely performed in Japan and is associated with an increase in maternal serious adverse effects. Japanese clinicians should adjust their practices of tocolytic treatment in accordance with the latest scientific evidence or make efforts to verify the effectiveness and safety of long-term tocolysis.

## Data Availability

The datasets analyzed during the current study are not publicly available for ethical reasons because the data are patient data; however, the datasets are available from the corresponding author on reasonable request.

## Abbreviation

ICD-10: International Classification of Diseases Tenth Revision
